# Single‐molecule mechanochemical characterization of *E. coli* pol III core catalytic activity

**DOI:** 10.1002/pro.3152

**Published:** 2017-03-16

**Authors:** M. Nabuan Naufer, David A. Murison, Ioulia Rouzina, Penny J. Beuning, Mark C. Williams

**Affiliations:** ^1^ Department of PhysicsNortheastern UniversityBostonMassachusetts02115; ^2^ Department of Chemistry and Chemical BiologyNortheastern UniversityBostonMassachusetts02115; ^3^ Department of Chemistry and BiochemistryOhio State UniversityColumbusOhio43210

**Keywords:** polymerization, polymerase, exonucleolysis, exonuclease, proofreading, DnaE, DnaQ, fidelity

## Abstract

Pol III core is the three‐subunit subassembly of the *E. coli* replicative DNA polymerase III holoenzyme. It contains the catalytic polymerase subunit α, the 3′ → 5′ proofreading exonuclease ε, and a subunit of unknown function, θ. We employ optical tweezers to characterize pol III core activity on a single DNA substrate. We observe polymerization at applied template forces F < 25 pN and exonucleolysis at F > 30 pN. Both polymerization and exonucleolysis occur as a series of short bursts separated by pauses. For polymerization, the initiation rate after pausing is independent of force. In contrast, the exonucleolysis initiation rate depends strongly on force. The measured force and concentration dependence of exonucleolysis initiation fits well to a two‐step reaction scheme in which pol III core binds bimolecularly to the primer‐template junction, then converts at rate *k*_2_ into an exo‐competent conformation. Fits to the force dependence of *k*_init_ show that exo initiation requires fluctuational opening of two base pairs, in agreement with temperature‐ and mismatch‐dependent bulk biochemical assays. Taken together, our results support a model in which the pol and exo activities of pol III core are effectively independent, and in which recognition of the 3′ end of the primer by either α or ε is governed by the primer stability. Thus, binding to an unstable primer is the primary mechanism for mismatch recognition during proofreading, rather than an alternative model of duplex defect recognition.

## Introduction

Replicative DNA polymerases are responsible for duplicating chromosomal DNA, which carries a large amount of information during cell division, and hence must be replicated with high accuracy to sustain life. They catalyze the addition of deoxynucleoside triphosphate (dNTP) units to the DNA backbone in DNA replication. The addition of the dNTPs occurs directly on the DNA template strand, and the base of the new dNTP is complementary to the base on the template strand. Since bases are added to the 3′ end of the nascent strand, the polymerization reaction must proceed in the 5′ to 3′ direction. The tertiary structure of DNA polymerase is such that the enzyme fits over the previously formed base pairs.^1^, ^2^ These bases must be paired correctly for the polymerase to adopt its functional conformation.^3^, ^4^, ^5^

DNA polymerase III (pol III) is the replicative DNA polymerase in *E. coli*.^6^, ^7^, ^8^, ^9^, ^10^, ^11^ It is an asymmetric dimer or trimer that synthesizes the leading and lagging strands simultaneously at the replication fork.^6^, ^7^, ^12^ A helicase unwinds the double‐stranded DNA (dsDNA) into two anti‐parallel template strands. After primase synthesizes the RNA primer, DNA pol III replicates the leading strand continuously and the lagging strand in Okazaki fragments in the 5′ to 3′ direction.^1^, ^6^, ^7^, ^8^ Pol III is composed of 10 subunits that coordinate leading and lagging strand synthesis. The core of the polymerase (pol III core) contains the polymerase subunit α, the proofreading exonuclease ε, and a subunit of unknown function θ.^13^, ^14^ The exact role of the θ subunit is still to be determined, but its presence increases the accuracy of pol III and it has been suggested to stabilize the interaction between α and ε.^15^, ^16^, ^17^ Although the dimer is asymmetric^7^, ^18^ to allow simultaneous polymerization of both template strands, the core of each branch consists of the same α ε θ complex.^13^ The polymerase subunit α is a C family DNA polymerase, a family that is found only in prokaryotes.^6^ DNA pol III has an extremely high catalytic rate, at 10^3^ bases/s,^13^, ^19^ and high fidelity, with error frequencies of approximately 10^−5^/bp without proofreading^13^, ^20^, ^21^ and 10^−8^/bp with proofreading.^13^ In the presence of the β clamp, which tethers the core protein assembly to its DNA substrate, α exhibits very high processivity.^13^, ^22^, ^23^

During replicative polymerization, tight coordination between the polymerization and exonucleolysis cycles is expected to exist, to permit efficient and faithful replication. It has been shown that mutations that lead to a loss in fidelity during *E. coli* replication are found in the *dnaQ* gene, which encodes the ε subunit.^24^, ^25^, ^26^ However, the molecular mechanism of the switching between the polymerase and exonuclease subunits is poorly understood. Elucidating the structure, function, and catalytic activity of these molecular motors is essential to understand the complex mechanisms of DNA replication. Here, we report a single‐molecule approach to manipulate these molecules and characterize the dynamics of pol III core polymerization and exonucleolysis.

We observe that the mechanical tension applied to the substrate DNA promotes the switching between exonucleolysis and polymerization functions, which agrees with previous single molecule studies on DNA polymerases Klenow Fragment, T7 gp5, and φ29 DNA polymerase.^27^, ^28^, ^29^ The force dependence of T7 polymerization velocity is modeled as a function of the free energy change involved in ssDNA‐dsDNA conversion.^27^ The kinetic scheme proposed for φ29 DNA polymerase describes the intramolecular primer transfer as a consequence of a conformational change in the φ29 pol ‐ DNA assembly induced by the applied tension on the DNA template.^28^ The key difference between pol III core and these polymerases is that the editing and polymerization activities of pol III core are carried out by distinct subunits, ε and α, respectively. Hence the primer transfer between the catalytic exo and pol domains occurs intermolecularly. In addition, the exo activity of isolated ε is similar to that of pol III core and ε is considered to be a highly efficient 3′‐5′ exonuclease, capable of functioning independently of α.^30^, ^31^ The source of this exonucleolytic editing specificity is found to be the greater melting capacity of a mispaired 3′ terminus for both isolated ε and pol III core.^32^, ^33^ According to previous bulk biochemical assays, the exonuclease activity of both ε and pol III core is more efficient with ssDNA.^32^ Furthermore, a two‐step kinetic scheme for the exonuclease reaction of isolated ε subunit suggests that the physiologically relevant substrate for the ε subunit within the holoenzyme complex is ssDNA at least three nucleotides in length.^33^

Here, we investigate the force dependence of pol III core polymerization and exonucleolysis. We are able for the first time to characterize these individual catalytic events on a single primer‐template DNA substrate. We propose a two‐state reaction scheme to describe the rate of force‐induced exo initiation. According to our model, pol III core bimolecularly binds at the primer‐template junction and subsequently transforms to an exo‐active conformation that is strongly affected by the applied template force. We show that this result is in quantitative agreement with the previously measured temperature‐dependence of exo‐activity. This analysis shows that the intermolecular switching of the primer between the polymerase and exonuclease subunits is a thermally driven process governed by destabilization of the primer‐template junction, rendering it more susceptible to exonuclease binding.

## Results

### Pol III core activity at constant force

**Figure 1:**
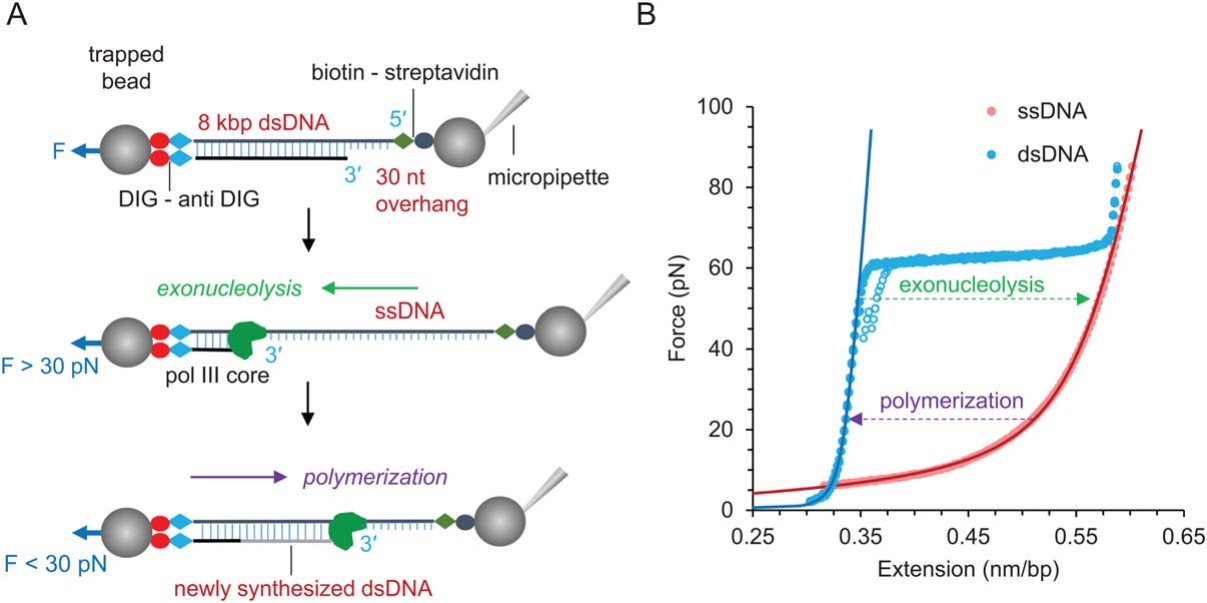
Schematic depiction of the single molecule experimental procedure using optical tweezers (not to scale). (A) Linearized pBACgus11 is ligated with a DIG‐dsDNA handle and a biotinylated oligonucleotide at its free ends, providing a single primer‐template junction for pol III core to bind. At forces above 30 pN, exonucleolysis is observed. Conversion from dsDNA to ssDNA upon the excision of nucleotides is registered as an increase in extension. Similarly, at forces below 30 pN incorporation of nucleotides due to polymerization is registered as a decrease in extension. (B) Force‐extension curves of dsDNA (blue) and ssDNA (red). Stretch and release are represented as solid and empty circles, respectively. The ssDNA is obtained by completely removing one strand of the dsDNA via exonucleolysis. Solid lines represent the theoretical polymer models: extensible wormlike chain^34^ for dsDNA and extensible freely jointed chain^35^ for ssDNA. The arrows show the direction of extension change of exonucleolysis and polymerization at constant force experiments.

We used optical tweezers to characterize the dynamics of pol III core activity at the single molecule level. Both polymerization and exonuclease activity were measured at constant applied tensions on a single DNA substrate. To do this, a single dsDNA molecule with a 3′ recessed end (∼30 nt) was attached by its covalently‐labeled free ends to polystyrene spheres, one held in an optical trap and the other immobilized on the end of a glass micropipette [Fig. 1(A)]. By gradually moving the micropipette, the applied mechanical tension and the extension of a single DNA molecule was measured. In the absence of protein, an approximately constant force phase transition, referred to as DNA overstretching, is observed [Fig. 1(B)].^36^ At the low‐salt conditions used in these experiments, this transition occurs at about 62 pN and represents a conversion of DNA from dsDNA to ssDNA as the DNA is destabilized by force and primarily peels from its free end.^37^, ^38^, ^39^, ^40^, ^41^, ^42^ The force‐extension profiles of ssDNA and dsDNA cross at ∼6 pN [Fig. 1(B)]. Pol III core activity was measured at forces greater than this crossover force, at which ssDNA is longer than dsDNA. Therefore, at constant forces below the melting transition, conversion between ssDNA and dsDNA is registered as an increase in extension due to pol III exonucleolysis (exo) and a decrease in extension due to pol III core polymerization (pol) activity [Fig 1(B)]. To measure pol III core activity, we introduced purified pol III core at fixed concentration to the flow cell containing a single DNA molecule captured between the beads as shown [Fig. 1(A)]. The DNA was maintained at a constant force through a feedback loop and the change in DNA length at constant force was determined as a function of time. The number of nucleotides incorporated or excised as a function of time was then obtained by dividing the observed change in extension by the expected change in extension at a given force accompanying the conversion of one single‐stranded nucleotide into its double‐stranded counterpart, as described in Materials and Methods.

**Figure 2:**
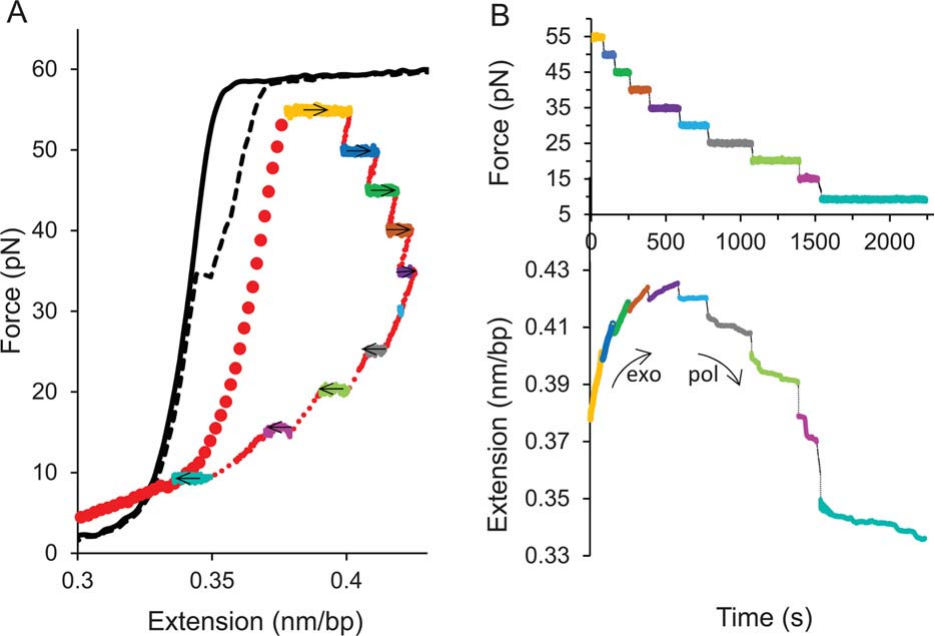
(A) Force‐extension curves of a bare dsDNA molecule (black) and a partially single‐stranded dsDNA molecule (red) in the presence of 0.2 μM pol III core. Changes in extension due to dsDNA‐ssDNA conversion at different constant forces are shown in the multicolor profile. The arrows represent the direction of the pol III core velocity, indicating exonucleolysis or polymerization. (B) Temporal trajectories of force (top) and change in extension (bottom) corresponding to the shown colors in (A).

**Figure 3:**
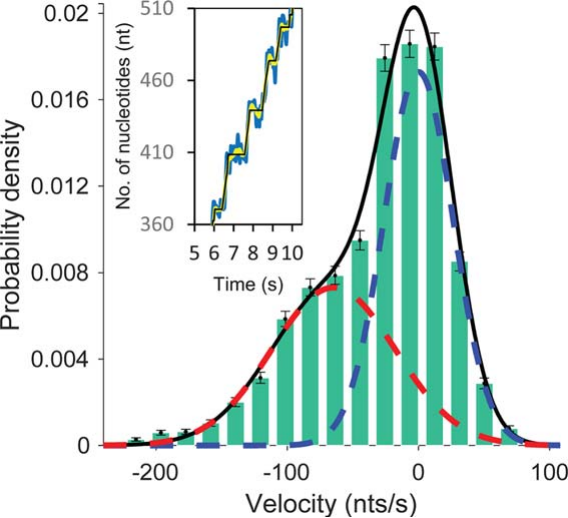
Representative bimodal Gaussian distribution of pol III core (0.2 μM) instantaneous velocities at 50 pN template tension determined from the moving average filter (yellow) shown in the inset. Zero (dashed blue) and nonzero (dashed red)‐peaked Gaussians represent the paused states and the moving states of pol III core, respectively. The peak position of the nonzero distribution represents the pol III core mean instantaneous velocity at 50 pN. Inset: Representative temporal trajectory (magnified) of the moving and paused states of pol III core. The pause detection (black, see Materials and Methods) is done using the moving average (yellow) of the extension data (blue).

**Figure 4:**
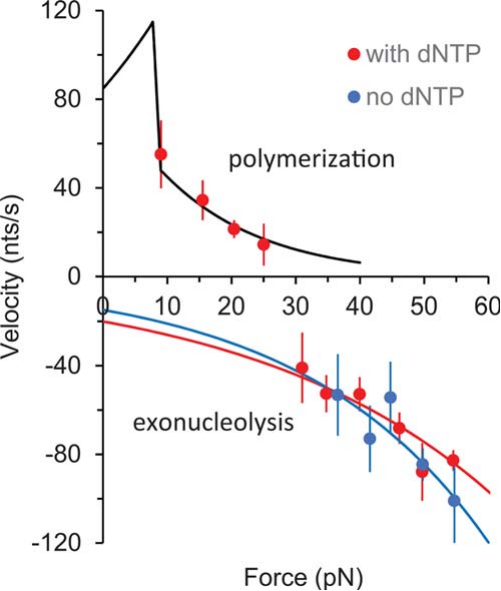
Force dependence of the pol III core instantaneous velocities. Positive velocities represent polymerization and negative velocities represent exonucleolysis in the presence (red) and absence (blue) of dNTPs. The force dependence of polymerization is fitted to the RCLM^43^ (black line) as described in the text, yielding 84.8 ± 5.7 nt/s as the zero‐force velocity (*v*_0_,_pol_). The exo force dependence is modeled as a simple exponential function of force, which yields *v*_0,exo_= −20 ± 5 nt/s, *d* = 0.11 ± 0.02 with dNTPs (red line) and *v*_0,exo_= −15 ± 10 nt/s, *d*= 0.14 ± 0.05 nm without dNTPs (blue line), where *d* is the force‐independent length change required for each exonucleolysis event. Error bars are standard errors of at least three independent measurements and uncertainties in the fitting parameters are from the standard deviation of the χ^2^‐minimized fit.

As shown in Figure 2, polymerization is observed at forces below 30 pN and exonuclease activity is observed at forces higher than 30 pN. From the measured extension vs. time trajectories, we obtained a distribution of instantaneous velocities for both exo and pol activity at several forces by applying a moving average filter as described in Materials and Methods. An example of a velocity distribution for exo activity at 50 pN is shown in Figure 3. Each distribution was fit as a sum of Gaussian functions, one at zero velocity and the other at finite velocity. The fit at finite velocity represents the distribution of instantaneous velocities characteristic of enzyme catalytic activity, while the zero‐velocity distribution represents instrument noise as well as protein fluctuations, and these data are not included in the velocity analysis. The average instantaneous velocities given by the mean of the Gaussian were obtained for each trajectory and averaged over at least three trajectories for all forces, and these averages for both exo and pol activity are shown in Figure 4. The instantaneous velocities are sensitive to the template tension, suggesting that the rate‐limiting step is force‐dependent. In addition, the sharp transition from pol to exo activity as a function of force also suggests that switching between these functions in the absence of force is expected to be thermally driven and the initiation of an exo event is facilitated by force.

### Force‐dependent polymerization and exonucleolysis velocities

The instantaneous velocities of T7 and Klenow Fragment DNA polymerases were previously modeled,^27^, ^29^ primarily attributing the force dependence to the activation enthalpy of converting n bases from the ss to the ds geometry. In that model, the projections of DNA segments along the direction of applied external force were determined “globally” by the change in the extension of ds and ss DNA extension as a function of force, averaged over thousands of bases. Therefore, that model does not distinguish the local orientations in the DNA segments inside the active site from the DNA segments further away from the active site. Consequently, Goel et al.^44^, ^45^ suggested a “local” model attributing the force dependence of polymerization velocity to the orientation change of two DNA segments neighboring the active site. It was also shown that large conformational changes in the pol‐template complex and a conserved active‐site geometry that induce a sharp kink at the 5′ end of the template during a catalytic pol event is a universal property shared by three families of polymerases.^44^ This model was further modified in Andricioaei et al.^43^ by restricting the orientations of these local DNA segments (Restricted‐Cone Local Model, RCLM) due to steric effects that were determined using molecular dynamic simulations on the *Thermus aquaticus* (Taq) DNA polymerase I complex. In this model, the instantaneous polymerization velocity (*v*(*F*)) is given by

(1) $$v(F)=v_{0}e^{-\Delta G(F)/k_{B}T},$$

where Δ*G* is the force‐dependent free energy contribution determined by the additional enthalpy and change in entropy associated with converting the two DNA segments from their “open” to “closed” forms, in the presence of force. For a given DNA segment **d**, the free energy contribution Δ*G*_d_ is given by

(2) $$\Delta G_{d}(F)=-\int_{0}^{F}\delta 〈\cos\theta 〉dF'.$$

Here 〈cos⁡θ〉 is the average angular orientation of a given DNA segment along the direction of force and is given by

(3) $$〈\cos\theta 〉=\frac{\cos\theta_{m}e^{\xi \cos\theta_{m}}-\cos\theta_{M}e^{\xi \cos\theta_{M}}}{e^{\xi \cos\theta_{m}}-e^{\xi \cos\theta_{M}}}-\frac{1}{\xi }.$$

The subscripts m and M refer to the minimum and maximum values used in RCLM, δ is the length of a given DNA segment, and $\xi =F\delta /k_{B}T$. Here *v*_0_ is the zero‐force instantaneous velocity, *k_B_* is the Boltzmann constant, and *T* is the absolute temperature. The reported θ and δ values for these two segments in the closed and open conformation in Andricioaei et al.^43^ were used in the force ranges less than and greater than 9 pN. Although this model used Taq pol I, which is an A family polymerase, and *E. coli* pol III α is a C family polymerase, another C family polymerase Taq pol III α was also shown to exhibit a bend of the template at the active site,^46^ suggesting that the basic features of this model are applicable in the present case.

The best fit of our data to the model of Andricioaei et al.^43^ yields *v*_0,pol_= 84.8 ± 5.7 nt/s (Fig. 4). Pol III core has a high velocity in the context of the complete replisome;^13^ however, the rate of replication of pol III core alone, which is the relevant comparison here, was measured in bulk biochemical experiments to be 20 nt/s.^47^ This significant difference in the velocities with and without the β clamp is likely due to the weaker association of pol III core with the substrate DNA in the absence of the β clamp. In contrast to single molecule assays, in bulk biochemical assays the catalytic rates are averaged over the paused states as well. Hence it is not surprising that the zero‐force velocity (*v*_0,pol_= 84.8 ± 5.7 nt/s) in our measurements is higher than the 20 nt/s rate observed in bulk biochemical experiments.

In the case of exo activity, there is not a similar previously applied model, so we will initially assume a simple exponential dependence on force given by

(4) $$v(F)=v_{0}e^{-Fd/k_{B}T}.$$

Here, *v*_0_ is the zero‐force exo velocity and d is the force‐independent length change required for each exonucleolysis event. The best fit yields, *v*_0_ = −20 ± 5 nt/s, *d* = 0.11 ± 0.02 nm with dNTPs and *v*_0_ = −15 ± 10 nt/s, *d* = 0.14 ± 0.05 nm in the absence of dNTPs. The value of d reflects an elongation of DNA that occurs during each exo event, which is likely the slightly extended state of the terminal base pair when it is positioned for cleavage during the exo rate‐limiting step. The fact that d (in both cases) is slightly less than the total change in DNA length [∼0.22 nm/bp, Fig. 1(B)] during an exo event supports this hypothesis.

Other alternative models, such as the model that describes φ29 pol and exo activity,^28^ are not consistent with our data. For φ29, the total velocity was presented as a sum of exo and pol activity, such that fluctuations between the states determined the total velocity. However, we do not observe a significant change in exo velocity in the presence and absence of dNTP, showing that pol activity is not present at forces that primarily induce exo activity. Also, as is shown below, in contrast to φ29, the time that pol III core spends on the DNA is much shorter than the switching time between pol and exo activities, such that in each processive event only one process is observed. Because pol and exo activity are accomplished by two separate proteins for pol III core, and ε by itself is an independent ssDNA nuclease,^33^ it is not surprising that these activities are so well separated.

### Concentration‐dependent pause times during exonucleolysis

**Figure 5:**
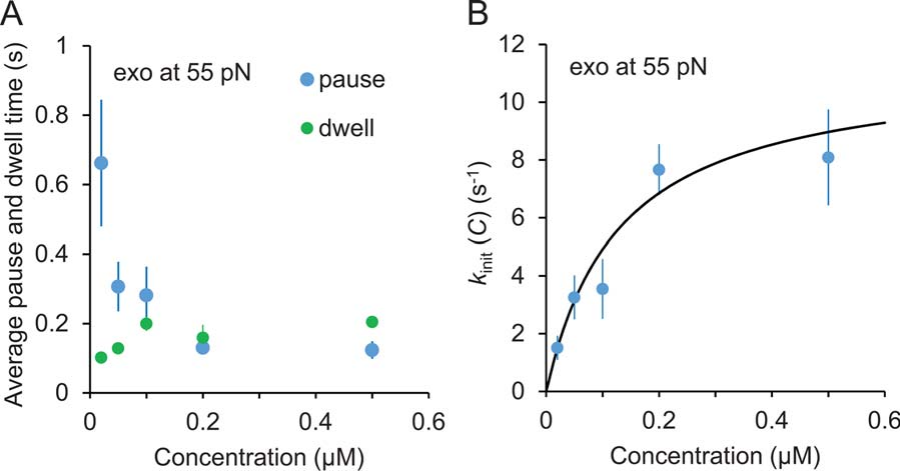
(A) Concentration dependence of pause (blue) and dwell (green) times during exo. Average pause time is significantly decreased with concentration. Dwell times do not exhibit a significant dependence on concentration. (B) Exo initiation rates, *k*_init_(C) as a function of pol III core concentration. The rate *k*_init_(C) is the reciprocal of the pause times shown in (A) and fitted to the two‐state model as described in the text [Eq. (6)]. The best fit yields the dissociation constant *K*_d_ = 0.13 ± 0.07 µM and transition rate to exo‐active state *k_2_* = 11.3 ± 3.1 s^−1^. Error bars are standard errors of at least three independent measurements and uncertainties in the fitting parameters are from the standard deviation of the χ^2^‐minimized fit.

The time spent during an exo event is measured as the dwell time (*τ*_d_). The time spent between two consecutive exo events is measured as the pause time (τ_p_), determined from the pause‐detecting trajectory shown in the inset of Figure 3. The reciprocal of the average *τ*_p_ is the exo initiation rate (*k*_init_) at a given force and concentration. The resulting concentration dependence of the pause and dwell times is given in Figure 5(A). Although *k*_init_ [Fig. 5(B)] initially increases with concentration, this rate saturates at high concentrations. To describe this concentration dependence, we propose the following kinetic scheme.

(5) $${E+\text{DNA}}_{n}\overset{k_{1}C}{\underset{k_{-1}}{⇄}}{E•\text{DNA}}_{n}\overset{k_{2}}{\underset{k_{-2}}{⇄}}{E•\text{DNA}}_{n}^{*}\overset{k_{\text{exo}}}{\to }{E•\text{DNA}}_{n-1}^{*}$$

Here, E is pol III core in the solution, C is the pol III core concentration, and DNA_n_ is the substrate that is the primer terminus at the primer‐template junction. The first step is the bimolecular pol III core binding, which is in pre‐equilibrium to the subsequent exo‐active ${E•\text{DNA}}_{n}^{*}$ state. The quantity *k*_2_ is the rate of pol III core transition to its exo‐active state, and *k*_−2_ is the rate of exo activity termination, due to dissociation that is determined by the dwell time measured below. As suggested by our measured force‐dependence of that rate (also discussed below) as well as by its strong temperature dependence measured in the previous studies,^32^, ^33^ exo initiation is rate‐limited by the requirement for destabilization of 2–3 bp at the primer terminus. Based on the pol III core structure,^48^ such destabilization is most likely followed by strand transfer of the 3′ end of the primer from the pol to exo catalytic site. This step is not accompanied by a net change in DNA construct length, and is therefore not directly observed in our experiment. However, conventional biochemical measurements of the exo initiation rates^31^, ^32^, ^33^ also suggest that DNA duplex destabilization is the rate‐limiting step of the process, supporting the notion that the subsequent strand transfer between the pol and exo sites occurs rapidly.

The next step is the catalytic exo activity that transforms ${E•\text{DNA}}_{n}^{*}$ to ${E•\text{DNA}}_{n-1}^{*}$.This process (*k*_exo_) is fast, and is not observed in the kinetics of exo‐initiation. This is because our analysis decouples the paused states from the moving states that are measured as a velocity (*v*_exo_), which ranges from 20 to 100 nt/s, depending on the force, as described in the previous section. For the proposed reaction scheme [Eq. (5)], the predicted exo initiation rate (*k*_init_) is given by,

(6) $$k_{\text{init}}(C)=\frac{k_{1}C}{k_{1}C+k_{-1}}k_{2}=\frac{1}{1+K_{d}/C}k_{2},$$

where,

(7) Kd=k−1k1

is the equilibrium dissociation constant for pol III core binding to the substrate. Note that because *k*_init_ is the reciprocal of the average pause time (1/*τ*_p_) for a single pol III core molecule to rebind to the substrate, this explicitly represents the on rate of an exo‐competent state and therefore, *k*_−2_ is disregarded in the equation. The best fit to the observed dependence of *k*_init_ on concentration [Fig. 5(B)], yields *K*_d_ to be 0.13 ± 0.07 µM and *k*_2_ to be 11.3 ± 3.1 s^−1^.

Equation (6) assumes that the first step of pol III core binding to DNA occurs in pre‐equilibrium to the slower catalysis initiation step *k*_2_, (*k*_−1_≫*k*_2_). According to our fitted values of *k*_2_ (11.3 ±3.1 s^−1^) and *K*_d_ (0.13 ± 0.07 µM), the bimolecular association rate *k*_1_ (*k*_−1_/*K*_d_) is much higher than *k*_2_ (*k*_1_>10^8^ M^−1.^ s^−1^), which is on the order of the diffusion rate (10^9^ M^−1.^ S^−1^). Thus, initial pol III core binding to the primer‐template junction is a nonspecific diffusion‐limited process, leading to the slower step of catalysis initiation, which subsequently results in either pol or exo activity, depending on the stability of the primer‐template junction.

### Force‐dependent pause times during exonucleolysis

**Figure 6:**
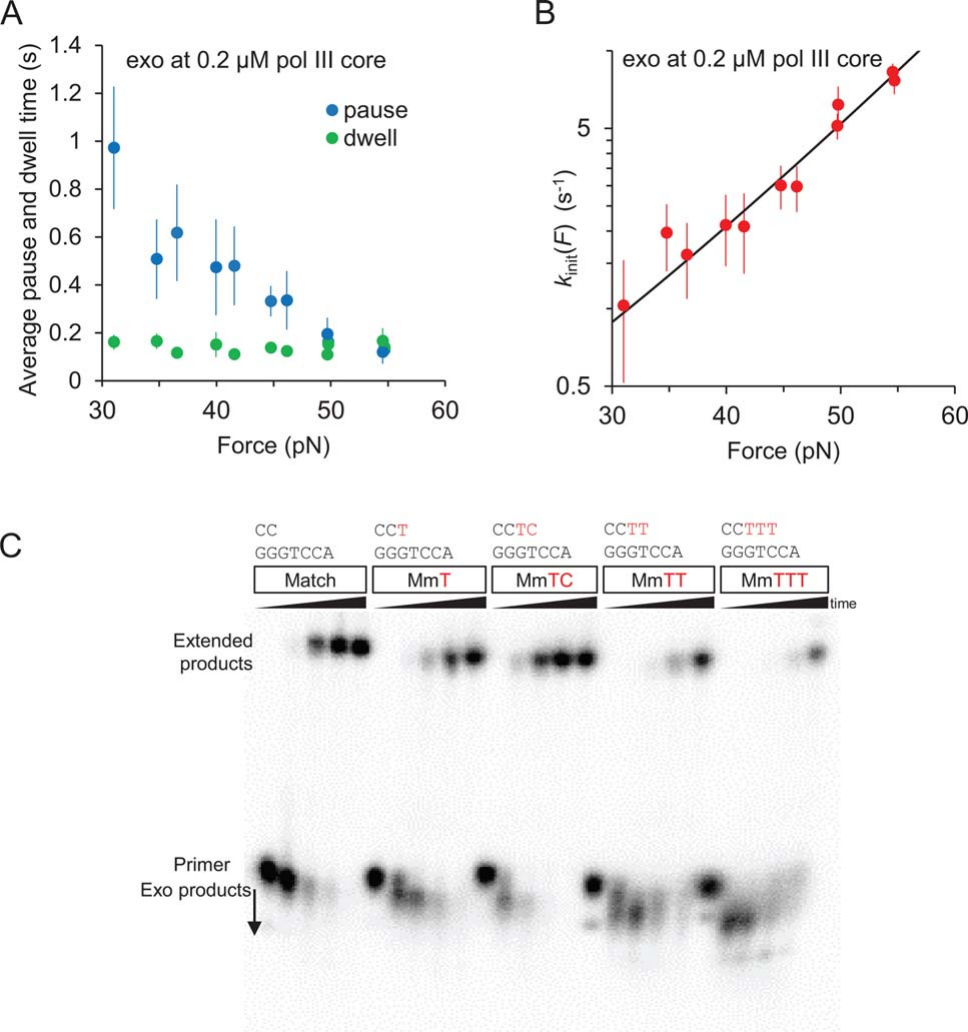
(A) Force‐dependence of average pause times (blue) and dwell times (green) during exo at 0.2 μM pol III core. Average pause times decrease with force. Average dwell times (green) exhibit insignificant force dependence. (B) Exo initiation rates, *k*_init_(*F*) as a function of force. The rate *k*_init_(*F*) is the reciprocal of the average pause times shown in (A) and is fitted to an exponential function of force as described in the text [Eq. (8)], which yields *n*_exo,init_ to be 1.84 ± 0.20 bp and *k*_F_ to be 17.3 ± 1.3 s^−1^. Here *n*_exo,init_ is the minimum number of base pairs required to melt at the primer‐template junction in order for the bound‐pol III core to transform to the exo‐competent conformation. The quantity *k*_F_ is the force‐independent attempt rate at the given concentration. Error bars are standard errors of at least three independent measurements and uncertainties in the fitting parameters are from the standard deviation of the χ^2^‐minimized fit. (C) Exonuclease activity of DNA pol III core complex is stimulated by base‐pair mismatches at the primer‐template junction. Fully‐extended polymerization products also decrease when 1, 2, or 3 noncomplementary bases are present at the junction. Reactions were quenched after 0, 1, 2.5, 5, and 10 min and analyzed by 12% denaturing polyacrylamide gel electrophoresis.

We observe a significant increase in the exo initiation rate *k*_init_ with increasing template tension [Fig. 6(B)]. The data in Figure 6 are measured at 0.2 μM pol III core concentration and therefore reflect the protein‐saturated value of *k*_init_ that is ∼*k*_2_. Thus, the observed force‐dependence of *k*_init_ primarily corresponds to the rate at which the bound protein‐DNA complex transforms into the exo‐active state. It has been shown that destabilization of the primer‐template junction increases the susceptibility of DNA to the exonuclease activity of pol III core and its exo subunit ε.^30^, ^32^, ^33^ Because template tension uniformly destabilizes all base pairs of stretched DNA,^49^ the force dependence of *k*_init_ is likely due to the increased probability of the duplex fraying, which can be described as^49^

(8) $$k_{\text{init}}=\;k_{F}e^{n_{\text{exo},\text{init}}(\Delta G^{†}(F)-\Delta G_{0}^{†})/k_{s}T}$$

Where,

(9) $$\Delta G^{†}(F)\;=\;\int_{0}^{F}x_{\text{ss}}(F')dF-x_{\text{ds}}(F')dF'.$$

Here *x*_ss_ and *x*_ds_ are the ss‐ and dsDNA extensions, respectively [Fig. 1(B)], and *k*_F_ is the maximum exo initiation rate on completely destabilized dsDNA or on ssDNA. This maximum exo initiation rate is expected to be reached at the melting force *F*_m_ (62 pN), at which the work performed by force to destabilize dsDNA, Δ*G^†^*(*F*), is equal to the free energy of bp melting in the absence of force $\Delta G_{0}^{†}$, 2.23 *k_B_T*. 50 The best fit yields *n*_exo,init_ to be 1.84 ± 0.20 bp and *k*_F_ to be 17.3 ± 1.3 s^−1^ µM^−1^. This value for *n*_exo,init_ is very similar to the number of mismatches required for optimal exo activity by the subunit ε observed in our bulk primer extension assay for pol III core, presented in Figure 6(C), and previous biochemical assays using isolated ε.^33^

### Pauses during polymerization

**Figure 7:**
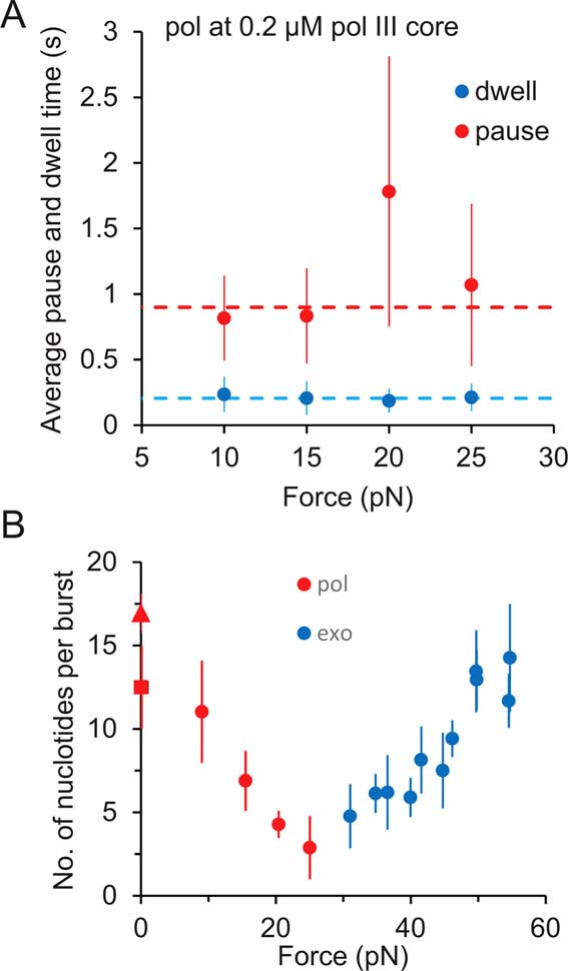
(A) Force‐dependence of average pause times (red) and dwell times (blue) during pol at 0.2 μM pol III core. Average pause and dwell times are independent of force during polymerization. The weighted average over all the forces of dwell time (dashed blue) is 0.20 ± 0.05 s and pause time (dashed red) is 0.90 ± 0.22 s shown by the dashed line. Error bars are standard errors of at least three independent measurements. (B) Average number of nucleotides (*N*) polymerized (red) or excised (blue) per single burst of catalytic activity of pol III core as a function of force. *N* was calculated as a product of measured velocities and dwell times of pol and exo activities, respectively. The red triangle is the N determined from the zero‐force velocity predicted from the RCLM (Fig. 4) and the red square is the zero‐force measurement reported in a previous biochemical study.^51^.

The observed pauses between consecutive catalytic pol bursts appear to be unaffected by the applied force within the accuracy of our measurement [Fig. 7(A)]. The weighted average of the pause time over all forces during pol activity is 0.90 ± 0.22 s. This suggests that in contrast to that observed for exo, initiation of a pol event is force‐independent.

### Dwell times for polymerization and exonucleolysis

The observed catalytic bursts during both pol (Fig. 7) and exo (Figs. 5 and 6) occur at short time scales (*τ_d,_*_pol_ = 0.21 ± 0.05 s, *τ_d,_*_exo_= 0.15 ± 0.05 s) that are independent of applied force or protein concentration (data not shown for pol). The reciprocal of the measured dwell time represents the termination rate of pol or exo activity (*k*_off_=1/*τ_d_* = 5–7 s^−1^) that is orders of magnitude higher than that observed for the pol III core complex in the presence of the β clamp.^13^, ^22^, ^23^ This significant difference in termination rates may account for the much weaker association of pol III core with the primer‐template junction in the absence of the β clamp. Because the observed *k*_init_ (C) (Fig. 5) for exo increases and saturates at high concentrations, this termination of catalysis primarily represents protein dissociation from the active conformation rather than intrinsic pausing during catalysis. Overall, this suggests that each exo event is associated with bimolecular protein binding to the primer‐template junction, which is followed by a complex transition to its exo‐active conformation.

### Number of processively catalyzed base pairs during polymerization or exonucleolysis

Figure 7(B) shows the number of nucleotides polymerized or excised per pol or exo activity burst, obtained as a product of the velocity (Fig. 4) and dwell time [Figs. 5(A) and 7(A)]. The number of catalyzed nucleotides vanishes at ∼25 pN for both exo and pol, indicating a transition from pol to exo [Fig. 7(B)]. Moreover, from the predicted zero‐force pol velocity (Fig. 4) and the measured dwell time [Fig. 7(A)], we can estimate the zero‐force value of the processivity to be ∼18 nt. This is consistent with previously measured bulk studies, in which pol III core was shown to incorporate 10–15 nucleotides before dissociating from the primer‐template junction.^51^

## Discussion

The force dependence of the catalytic functions of polymerases from bacteriophage φ29 and T7 as well as the *E. coli* polymerase Klenow Fragment (KF) has been previously studied using single‐molecule stretching experiments.^27^, ^28^, ^29^ Our single molecule results for the force dependence of pol III core catalytic activity qualitatively agree with the previously studied polymerases to the extent that force inhibits or facilitates polymerization and exonuclease activity, depending on the force relative to the 6 pN crossover point. However, pol III core is a weakly processive polymerase that only incorporates ∼20 nucleotides before dissociating from the primer‐template junction.^51^ This weak processivity of pol III core imposes an additional challenge on studying its function and characterizing its activity, especially at the single‐molecule level, demanding high resolution data acquisition. In this study we have successfully probed the force dependence of both the polymerase and exonucleolysis functions of pol III core, and by modeling the force dependence of the observed pol and exo velocity and pausing, we obtain significant new insights into how these processes are regulated.

One major difference between pol III core and the previously studied T7 and φ29 polymerases is that pol III core is a multienzyme assembly in which the pol and exo domains are different subunits, α and ε, which can function independently even when not part of core.^33^ In fact, a recent study estimated that the distance between the polymerase and exonuclease active sites in pol III core is greater than 7 nm.^48^ Therefore, for the exo activity to initiate after pol III binding to duplex DNA, the 3′ end of the primer strand has to move from the pol catalytic site in α into the exo catalytic site in ε. The length change associated with the force dependence of the exo initiation rate *k*_init_(F) is only ∼0.44 nm [2 bp × ∼0.22 nm/bp, where ∼0.22 nm/bp is the extension change associated with melting one DNA bp at F >30 pN, Fig. 1(B)]. Because this length change is much smaller than the distance between the active sites of α and ε, α binding to the template must be disrupted to allow ε to bind to the frayed strand.

The structural autonomy of the two catalytic domains of pol III core may result in more independent functions between the two proteins. In the case of T7 and φ29 DNA polymerases, duplex DNA binds to the polymerase active site and exonucleolysis is facilitated via an intramolecular transfer through several intermediate steps.^28^, ^52^, ^53^ Although the applied force favors exo and suppresses the pol activity of φ29, the underlying mechanochemistry is significantly different from pol III core. Specifically, in contrast to pol III core, the dwell time of φ29 is much longer than its time of switching between pol and exo activities. The average velocity of φ29 catalysis appears to be a continuous function of the force with several fine features suggesting intermediate steps in the pol to exo switching process.^28^ In contrast, ε by itself has been shown to be an ssDNA exonuclease, and the catalytic activity of ε is similar on ssDNA and mispaired primer termini. The ε subunit preferentially binds ssDNA, whereas α binds both ssDNA and dsDNA and prefers a primer‐template junction.^30^, ^54^ In addition, a recent NMR study showed that a primer destabilized due to mismatches increases the propensity of the mismatch to reach the ε subunit, enabling ε to correct for the mismatches in a passive manner.^55^ Thus, a simple model for the regulation of exo and pol activity is based primarily on the preferential binding of each protein for specific DNA substrates. Because α binds strongly to a stable primer‐template junction, while ε binds strongly to ssDNA free ends, the switch between pol and exo is determined by the stability of the primer‐template junction, which is reduced upon the application of large forces or at high temperatures. These conditions, as well as mismatches at the primer‐template terminus, induce a shift from pol to exo activity, and these activities will be considered independently below.

### Force‐dependent instantaneous velocity of pol III core exonucleolysis

We have modeled the force‐dependence of the pol and exo instantaneous velocities of pol III core as independent processes. Here, for the first time we were able to directly measure the instantaneous exo velocity on a properly paired dsDNA substrate uniformly destabilized by a stretching force parallel to the DNA axis. Interestingly, this velocity ranges between 40 and ∼110 nt/s as the stretching force increases from 30 to 55 pN (Fig. 4). Because at 55 pN the dsDNA is very close to its melting force of 62 pN, we approximate the latter measurement as the maximum catalytic exo rate of pol III core. The observed velocities at higher forces (∼100 nt/s) are independent of the pol III core concentration (data not shown) and are also not strongly facilitated by the force. The exponential dependence of the observed exo velocities yields *v_0,_*_exo_ = 20 ± 5 nt/s and *d*= 0.11 ± 0.02 nm with dNTPs and *v_0,_*_exo_ = 15 ± 10 nt/s and *d* = 0.14 ± 0.04 nm without dNTPs. The independence of both parameters within uncertainty to the presence of dNTPs suggests that ε acts independently from the polymerase α, even in the context of pol III core. The length change required during a rate‐limiting step of processive catalytic excision is significantly smaller than that of the length change required for exo initiation [2 bp ∼ 0.44 nm, Fig. 6(B)], which is the rate‐limiting step for the exo process. Thus, while exo initiation is relatively slow and strongly dependent on the primer‐template terminus stability, the instantaneous exo velocity is about ∼10–100 fold faster and depends weakly on the base pair stability. This result indicates that the catalytic excision by itself is likely not strongly affected by the presence of mismatches or the DNA sequence at the junction.

### Force‐dependent instantaneous velocity and pausing of pol III core polymerization

The instantaneous pol velocity is strongly affected by the applied force (Fig. 4). However, the dwell times during pol are approximately force‐independent [Fig. 7(A)]. The zero‐force pol velocity (*v*_0,pol_ *)* was found to be 84.8 ± 5.7 nt/s based on the model of Eqs. (1) and (2). Therefore, the predicted number of nucleotides synthesized by pol III core at zero force during an average dwell time of 0.21 s is ∼18 nt [Fig. 7(B)]. Pauses during consecutive pol bursts are relatively longer than in exo and are poorly or not at all affected by the applied force. We find the average pause over all forces to be 0.90 ± 0.22 s at 0.2 μM pol III core. Thus, in strong contrast to the exo activity, the instantaneous catalytic velocity of the pol activity is strongly affected by force, while the initiation of a pol event is insensitive to the applied force. This result is consistent with the fact that pol binds strongly to a stable primer‐template junction, the presence of which does not depend strongly on force at pol‐competent forces. In contrast, exo requires a highly force‐dependent destabilized primer‐template junction for initiation, as discussed below. However, once initiation of exo occurs, exo activity can proceed without further requirements for base pair destabilization.

### Two‐step exonucleolysis initiation from concentration‐dependent measurements

**Figure 8:**
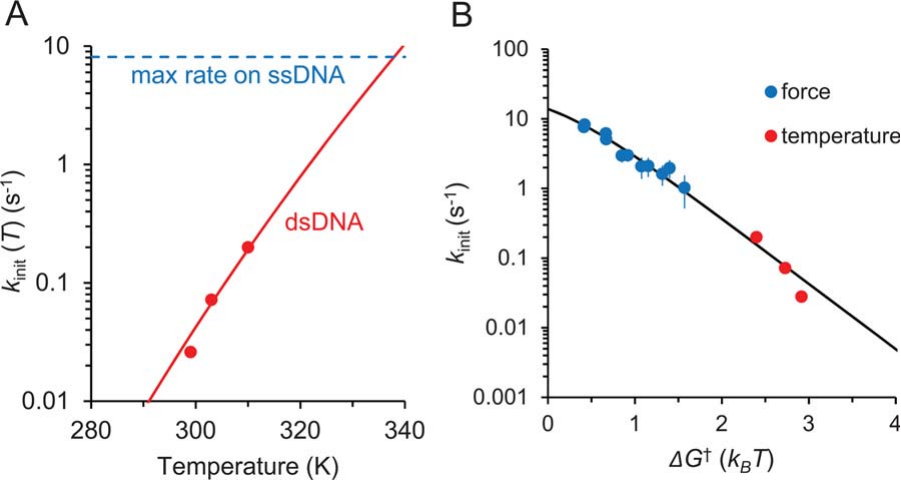
(A) Temperature‐dependence of the exo initiation rate as reported in Brenowitz et al.^32^ Red circles are the reported exo initiation rates on a G‐C paired primer‐template junction, scaled by a factor of 0.02 to match the experimental conditions in this study (see Discussion). The reported data is fit to an Arrhenius function as described in Eq. (11). The best fit (red line) yields *n*Δ*H*_0_ = 47 *k_B_T*. The dashed blue line at 8.1 s^−1^ is the maximum rate reported, which is observed on ssDNA at 310 K. (B) The rate *k*_init_ determined from force‐ (blue) and temperature‐ (red)^32^ dependent measurements as a function of total free energy Δ*G* ^†^(*F,T*) required to destabilize terminal dsDNA. The global fit is from the expression *k*_0_exp[‐*n*_exo,init_ Δ*G* ^†^/*k_B_T*]/(1 + exp[‐*n*_exo,init_ Δ*G* ^†^/*k_B_T*]), that describes the probability of destabilizing *n*_exo,init_ terminal base pairs as a function of Δ*G* ^†^(*F*,*T*). Here Δ*G* ^†^ (*T*) = Δ*H*_0_ ‐ *T*Δ*S*_0_, where Δ*H*_0_= 17 *k_B_T* and *T*Δ*S*_0_ = 0.0471*T k_B_T*, as described in the text. Δ*G* ^†^ (*F*) is given by Eq. (8). The best fit yields *n*_init,exo_ = 2.16 ± 0.13 bp and *k*_0_ = 27.9 ± 1.9 $s_{.}^{-1}$

We model the observed concentration dependence of exo‐initiation rates using the proposed reaction scheme shown in Eq. (5). The pol III core‐saturated value for *k*_init_≈*k*_2_ (11.3 ± 3.1 s^−1^ at 55 pN), the rate at which an exo‐active state is achieved, can be compared to the rate of a single exo‐cut by pol III core measured previously using bulk biochemical assays.^32^ After correcting the results in Brenowitz et al.^32^ (as discussed below) to match our experimental conditions, we find that *k*_init_ for ssDNA at 310 K is ∼8.1 s^−1^ [dashed line, Fig. 8(A)]. This represents the maximum value that characterizes *k*_init_ for a completely destabilized DNA substrate, obtained by increasing force or temperature. The similarity of this result to our single molecule measurements at high forces (55 pN) approaching the DNA melting force (62 pN) brings confidence that we are measuring the same process (Fig. 6). Furthermore, we measured *K*_d_ to be 0.13 ± 0.07 μM, which agrees with the previously measured values of 0.14–0.46 μM^30^, ^32^ for comparable solution conditions.

### Exonucleolysis initiation is determined by primer‐template junction stability

We model the force dependence of the exo‐initiation rate as a function of the complete work done by force to transform DNA from its double‐ to single‐stranded form as described in Eqs. (8), (9).^49^ We find that at least two base pairs (*n*_exo,init_=1.84 ± 0.20 bp) are required to be destabilized through thermodynamic fluctuations to enter the exo‐active state of pol III core. As shown in our primer‐extension assay with pol III core [Fig. 6(C)] and as demonstrated by Miller et al.^33^ for isolated ε, there is a significant increase in exo activity when the number of mismatches at the primer‐template terminus is altered from one to two, in remarkable agreement with our single‐molecule results.

At concentrations above the pol III core‐DNA binding *K*_d_, the force dependence measured is determined primarily by *k*_2_. Thus, simple diffusion‐limited bimolecular binding of the enzyme to its DNA substrate occurs in pre‐equilibrium to exo initiation. The exo initiation rate is strongly affected by dsDNA stability, leading to a strong force and temperature dependence of *k*_init_. If pol III core exo activity is primarily rate‐limited by the destabilization of the primer‐template terminus, our force‐dependent measurements should be comparable to the previously measured temperature dependence of its activity.^32^ The force‐dependence of exo initiation *k*_init_(F) in our study is analogous to the rate of single nucleotide excision on matched GC‐terminated and single‐stranded DNA reported in Brenowitz et al.^32^ To compare these values to those measured here, we scale the reported parameters of the Michaelis‐Menten excision reaction (*V*_max_/*K*_m_) to determine *k*_init_(T), where,

(10) $$k_{\text{init}}(T)=\left(\frac{V_{\max}}{K_{M}}\right)\times \left(\frac{K_{M}}{C}\right).$$

Here, we took into account that the *K*_M_ is ∼400 nM as measured in the same work at 2 nM pol III core concentration (C), and shown in Figure 8(A).^32^ Furthermore, we modeled *k*_init_(T) as an Arrhenius function analogous to the Eq. (8) where,

(11) $$k_{\text{init}}\left(T\right)=k_{T}e^{-\;\frac{n_{exo,init}\Delta G^{†}(T)}{k_{B}T}}=k_{T}e^{-\;\frac{n_{\text{exo},\text{init}}\left(\Delta H_{0}-T\Delta S_{0}\right)}{k_{B}T}}$$

Here Δ*G* ^†^(*T*), Δ*H*_0_ *and* Δ*S*_0_ are the free energy, enthalpy and entropy, respectively, of a single bp melting at a reference temperature *T*, and *n*_exo,init_ is the number of base pairs melted during the rate‐limiting step of exo‐initiation. The best fit yields *n*_exo,init_Δ*H*_0_ to be 47 *k_B_T*. Because the enthalpy of a single base pair melting, Δ*H_0_*, is ∼17 *k_B_T*,^56^ *n*_exo,init_, the minimum number of destabilized base pairs required in the rate‐limiting step for exo, can be estimated to be ∼2.5 bp. Furthermore, a global fit [Fig. 8(B)] to both the force and temperature dependence as a function of the free energy required to destabilize dsDNA, determined using the same values, yields *n*_init,exo_ = 2.16 ± 0.13 bp. This agrees remarkably well with the value determined for *n*_exo,init_ (1.8 ±0.2 nt) obtained only from our force‐dependent measurements. The excellent compatibility between the force and temperature dependence of the pol III core exo activity confirms that the rate limiting step *k*_2_ is primarily dependent on the stability of the primer‐template terminus. Furthermore, the exo catalytic functions of pol III core and ε are shown to be similar once scaled with their appropriate *K*_d_ values.^32^ This suggests that the requirement for destabilization at the primer‐template terminus for the onset of exo activity by either ε or pol III core are very similar. However, pol III core alters the geometry of bound ε, in which the 3′ end of the primer strand is required to be displaced a significant distance to the catalytic site of ε to trigger the onset of exo activity.

## Conclusions

Taken together, our results support a model in which the pol and exo activities of pol III core are effectively independent and the stability of the primer‐template junction determines the selection between α and ε binding to the 3′ end of the primer strand. Once pol III binds in a pol‐ or exo‐competent conformation, enzymatic activity proceeds with relatively high velocity of 10–100 nt/s (Fig. 4). Despite these high catalytic rates for the pol‐ and exo‐domains, the processivity of pol III core remains low at 10–20 nt [Fig. 7(B)], as both catalytic events occur via short ∼0.2 s bursts [Fig. 7(A)]. These bursts are interrupted by pol III core dissociation from the primer‐template junction. Re‐initiation of exo activity requires re‐binding of pol III to the primer‐template junction from solution, followed by the slower melting of ∼2 bp at the primer terminus. This is then followed by the much faster transfer of the 3′ end of the primer strand to the pol III exo site. The force dependence we measure for exo initiation closely matches the previously measured temperature dependence of the same process.^32^ This result supports a model in which mismatch recognition during proofreading is determined by primer template duplex end stability, rather than a model of duplex defect recognition.^32^, ^33^ Overall, the independent nature of the pol and exo states as well as frequent dissociation of pol III from its substrate are expected to allow stronger regulation of these processes by other factors involved in *E. coli* replication.

## Materials and Methods

### Pol III core expression, purification, and biochemical analysis

Wild‐type DNA pol III core was expressed from the plasmid pET16b‐dnaE‐holE^H^‐dnaQ, which features a His‐tag on the θ subunit (a generous gift from Mark Sutton, Univ. at Buffalo), as described.^57^ Core was purified from a cell pellet harvested from 1 L of culture and stored at −80 °C. The cells were thawed on ice and lysed by sonication. Clarification was carried out by centrifugation at 12000 x g for 1 h at 4 °C. The supernatant containing soluble proteins was passed through a 0.45 μm filter before loading on a 5‐mL His‐Trap HP column (GE Healthcare) equilibrated with buffer HisA [20 mM HEPES; 500 mM NaCl; 50 mM imidazole; 10% glycerol, pH 7.5]. Bound protein complex was eluted using buffer HisB [20 mM HEPES; 500 mM NaCl; 300 mM imidazole; 10% glycerol, pH 7.5]. Fractions containing the desired protein were pooled and diluted 10‐fold using buffer HeparinA [50 mM HEPES (pH 7.5); 0.1 mM EDTA; 10% glycerol; 1mM DTT] before loading onto a 5‐mL Hi‐Trap Heparin HP column (GE Healthcare) equilibrated with HeparinA. Bound proteins were eluted by addition of buffer HeparinA + 1 M NaCl in a linear gradient. Fractions containing intact core complex were pooled and diluted 6‐fold with buffer HydroxyA [50 mM HEPES (pH 7.5); 150 mM NaCl; 1 mM DTT; 10% glycerol] and loaded onto a 5‐mL hydroxyapatite column (BioRad Bioscale Mini CHT Type 1, 5 ml, 40 mm cartridge) equilibrated with buffer HydroxyA. Bound protein was eluted with buffer HydroxyB [200 mM sodium phosphate (pH 6.5); 150 mM NaCl; 1 mM DTT; 10% glycerol] in a step gradient. Fractions containing protein complex were pooled and dialyzed overnight at 4 °C against 2 L of storage buffer [30 mM HEPES (pH 7.5); 100 mM NaCl; 0.5 mM EDTA; 2 mM DTT; 20% glycerol]. Protein purity was determined by SDS‐PAGE, proteins were quantified by Bradford assay, and purified complex was stored at −80 °C.

Primer extension assays were carried out as described previously^58^ using 32P‐labeled primers annealed to 61‐mer template. Reactions contained a final concentration of 25 nM DNA polymerase, 100 nM primer/template DNA, 100 µM dNTPs, 7.5 mM MgSO_4_, 30 mM HEPES (pH 7.5), 20 mM NaCl, 2 mM DTT, 1% (w/v) bovine serum albumin, and 4% glycerol. Reaction products were separated by denaturing 16% polyacrylamide gel electrophoresis and analyzed by phosphorimaging. The template sequence is 5′‐ ggttactcagatcaggcctgcgaagacctgggcgt ccggctgcagctgtactatcatatgc; the primer sequences are Match: 5′‐ gcatatgatagtacagctgcagccggacgcc; MmT: 5′‐ gcatatgatagtacagctgcagccggacgcct; MmTC: 5′‐ gca tatgatagtacagctgcagccggacgcctc; MnTT: 5′‐ gcatatga tagtacagctgcagccggacgcctt; MmTTT: 5′‐gcatatgatagta cagctgcagccggacgccttt.

### Single molecule DNA constructs

Either a 38.5‐kbp λ DNA or an 8.1 kbp pBacgus11 DNA were used in the single molecule stretching experiments. The 48.5‐kbp linear λ DNA (Roche) with 12‐nt 5′ overhangs at both the termini was digested with ApaI (New England Biolabs, NEB). The 5′ overhang of the resultant 38.5‐kbp substrate was filled‐in with KF in the presence of dGTP, dATP, biotin‐14‐dATP, and biotin‐14‐dCTP (NEB). At the opposite end a biotinylated oligonucleotide (5′‐bbCTCbTCTCbTCT CTTCTCTCTTCTCTTGGCC‐3′, Integrated DNA Technologies, IDT) consisting of a 3′ end complementary sequence to the ApaI‐digested site was ligated with T4 DNA ligase. The 8.1 kbp construct was created by first linearizing the pBacgus11 (a gift from Borja Ibarra) dsDNA vector (8041 bp) with BamHI and SacI (NEB). A digoxigenin (DIG) labeled dsDNA handle with a complementary sticky end to the BamHI sequence was generated as described.^28^ A biotinylated oligonucleotide (5′‐bbCTCbTCTCbTCTCTTCTCTCTT CTCTTGGCCAGCT‐3′, IDT) with a 3′ end complementary to SacI sequence, and the dsDNA DIG‐handles were then ligated to their complementary positions at the linearized pBacgus11 DNA using T4 DNA ligase. In both of the biotinylated oligos, the position of biotin is indicated by b.

### Single molecule optical tweezers experiments

We used optical tweezers to induce tension in single DNA molecules and thereby facilitate the pol III core activity. Here, a single DNA molecule was attached by its labeled ends to derivatized polystyrene spheres. The 38.5‐kbp λ DNA biotinylated at both the termini or 8.1‐kbp pBACgus11 DNA, with biotinylated and DIG handles ligated at its respective termini, were tethered at the ends with streptavidin or streptavidin and anti‐digoxigenin coated beads. One bead was immobilized by a glass micropipette attached to a flow cell while the other was held in a dual beam optical trap. By moving the glass micropipette attached to the flow cell, the DNA molecule was stretched and the force required to extend the DNA molecule was measured. The solution surrounding a single DNA molecule was replaced with pol III core (20, 50, 100, 200, and 500 nM) diluted in the reaction buffer, 50 mM HEPES at pH 7.5, 25 mM Na^+^, 10 mM MgCl_2_, 5 mM DTT and 1% BSA. In addition, 0.3 mM (each) dNTP were added to the experiments with dNTP at 0.2 μM pol III core. For some experiments, the exo activity of T7 DNA polymerase (NEB) was used initially to create a partial ssDNA substrate, then exchanged for pol III core. Data were collected at constant forces at 25 Hz, in which a detected change in the tension of the DNA substrate is compensated with a change in extension via a force feedback loop. The conversion between dsDNA‐ssDNA upon exonucleolysis or polymerization, at constant DNA tensions, is registered as a change in extension as a function of time.

### Single molecule data analysis

The extension‐time trajectory was filtered with a moving average window of 8 Hz. The change in extension was converted to number of replicated or excised nucleotides by dividing the observed distance change by the expected change in extension at a given force accompanying the event of a single nucleotide incorporation. Theoretical polymer models, extensible worm like chain^34^ for dsDNA, and extensible freely jointed chain^35^ for ssDNA, were used to calculate the expected change in extension at a given force. Polymerization or exonucleolysis velocity distributions were obtained from the moving average trace and fit to a bimodal Gaussian to find the instantaneous catalytic rate. Pauses were captured in the filtered trace by setting the consecutive events less than a cutoff of $5/\sqrt{w}$ nt, to their mean values using a custom MATLAB code. Here 5 nt is the experimental noise and *w* is the number of data points included within the chosen window. We tested our algorithm with simulated data and the accuracy of recovered results were >90%, when the random noise level was set as 5 nt.
